# Artificial intelligence in microsurgery and supermicrosurgery training within plastic surgery: A systematic review

**DOI:** 10.1016/j.jpra.2025.07.010

**Published:** 2025-07-19

**Authors:** Cheuk Ying Kyleen Kiew, Anuska Shah, Michalis Hadjiandreou, Georgios Pafitanis

**Affiliations:** a Faculty of Medicine, Imperial College London, Ayrton Road, South Kensington, London, United Kingdom; b Department of Plastic and Reconstructive Surgery, St Andrew’s Centre, Broomfield Hospital, Mid and South Essex NHS Foundation Trust, Court Road, Chelmsford, United Kingdom; c Department of Plastic and Reconstructive Surgery, The Royal London Hospital Microsurgery Unit, Bart’s Health NHS Trust, Whitechapel Road, London, United Kingdom

**Keywords:** Microsurgery, Training, Education, Artificial intelligence, Supermicrosurgery

## Abstract

**Introduction:**

Artificial Intelligence (AI) is increasingly integrated into plastic surgery. Technical complexities of microsurgery and supermicrosurgery demand incorporation of AI in facilitating steep learning curves. Although simulation models exist, many provide inadequate preparation and fidelity. AI has potential to complement trainee efforts in skill acquisition by improving training efficacy. This systematic review aims to present and evaluate applications and future of AI in microsurgery and supermicrosurgery plastic surgery training.

**Materials and Methods:**

PubMed, Embase, Scopus and Google Scholar were searched following PRISMA guidelines. Studies on AI applications on microsurgery and supermicrosurgery training within plastic surgery were included. Two independent reviewers screened the literature, extracted data and assessed risk of bias using ROBINS-I tool. AI model architecture and motion, eye and instrument tracking parameters and narrative data synthesis were captured on Microsoft Excel. (PROSPERO ID: CRD42025607695).

**Results:**

Five articles were included. Two articles developed automated microsurgical skill classification datasets with force-based surgical glove model achieving above 95 % accuracy. Two AI applications used eye tracking parameters namely, blink rate, pupil dilation and gaze coordination to assess proficiency and workload. Path length, mean velocity and jerk curvature were accurately analyzed by AI models with instrument and hand tracking. All studies reported moderate risk of bias.

**Conclusion:**

This is the first systematic review addressing AI applications in microsurgery and supermicrosurgery training. AI models tracking eye, hand and instrument motion for microsurgical skill assessment demonstrated accuracy and precision, enabling real-time monitoring of proficiency and workload, which are scalable, objective methods for improving training and outcomes.

## Introduction

Microsurgery and supermicrosurgery training have evolved over time with a focus on competency-based training and assessments on validated simulation models.^1^ This is due to steep learning curves, limited hands-on opportunities in operating theatres, constraints in training hours and ethical considerations of training, creating an inconsistent, high-pressure and unpredictable training environment.^2^, ^3^, ^4^, ^5^ To address such challenges, several animal, synthetic and human cadaveric models have been implemented to the microsurgery curriculum to supplement clinical exposure.^6^ However, live feedback on potential areas for improvement is restricted to a senior surgeon’s availability and willingness to teach, with feedback often being subjective, time-consuming to provide and difficult to implement.

Artificial Intelligence (AI) has emerged as a transformative tool offering novel solutions to longstanding challenges in surgical education.^7^ Through advancements in machine learning (ML), computer vision, and deep learning algorithms, AI has the ability to enhance microsurgical training by providing objective skill assessment, personalized feedback and automated detection of technical errors.^8^^,^^9^ Despite its potential, AI-driven approaches remain largely unexplored with existing literature focusing on biological and non-biological simulation models and limited studies addressing its applications and efficacy.^10^

This systematic review aims to evaluate the existing evidence on AI applications in microsurgery and supermicrosurgery training by analyzing the current AI-driven training models, their performance metrics, and their potential for future integration in microsurgery and supermicrosurgery training.

## Methods

**Figure 1:**
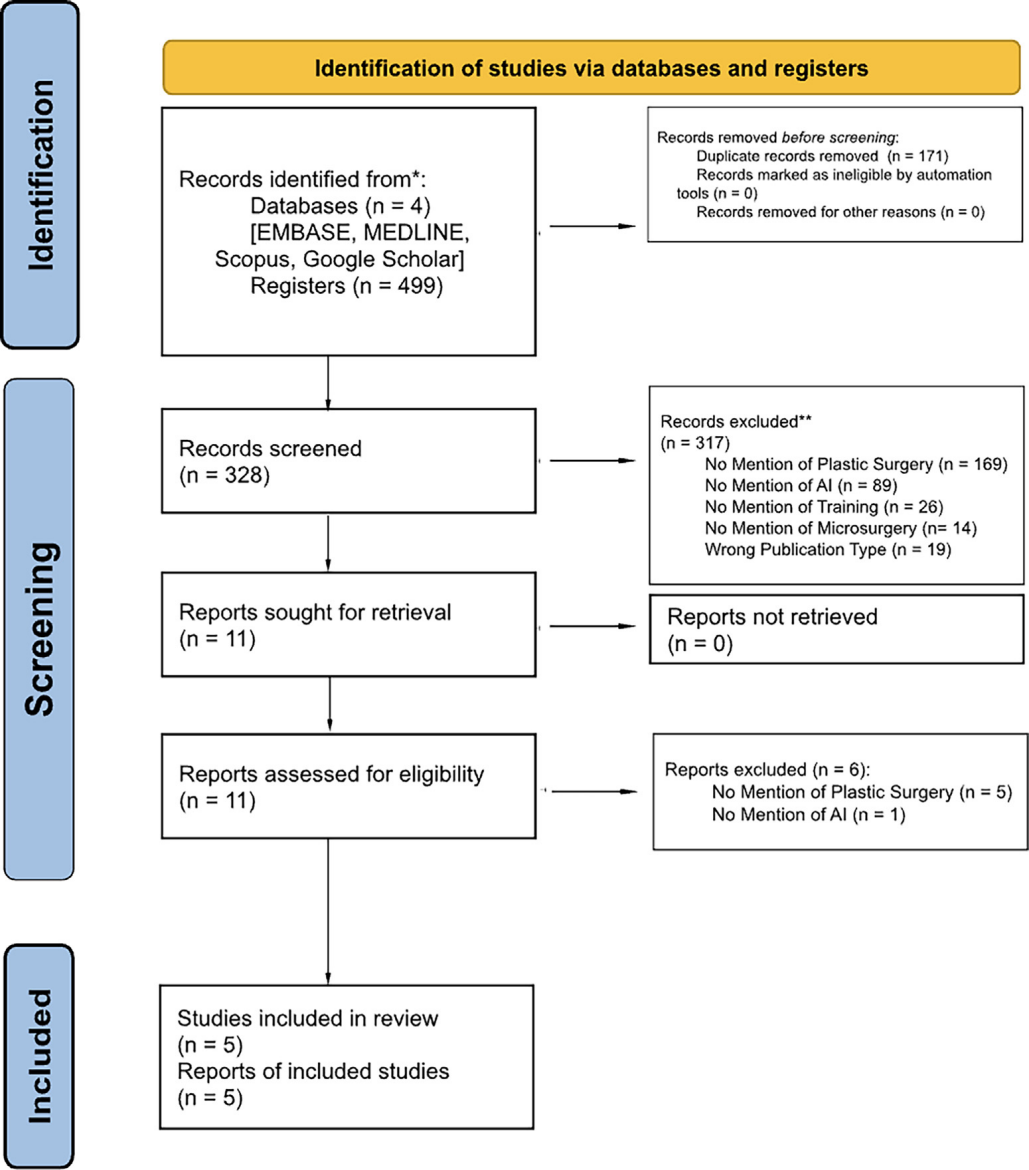
Systematic review algorithm, employing the Preferred Reporting Items for Systematic Reviews and Meta-Analyses (PRISMA) guidelines in the Pubmed, Embase, and Scopus, Web of Science, Google Scholar, and the Cochrane Library databases.

This systematic review adhered to the Preferred Reporting Items for Systematic Reviews and Meta-Analyses (PRISMA) guidelines.^11^ See Figure 1. The protocol for the study was prospectively registered on PROSPERO (PROSPERO ID: CRD42025607695).

### Search methods

A thorough literature search was conducted on PubMed, Embase, Scopus, Web of Science, Google Scholar, and the Cochrane Library for studies published from inception to 2025. The search strategy included MeSH terms, and Boolean operators, of which includes (microsurgery OR microsurgical OR supermicrosurgery) AND (training OR education OR curriculum OR simulation) AND (artificial intelligence OR neural networks OR machine learning) AND (plast*).

### Selection criteria

Studies describing AI training models for microsurgery or supermicrosurgery training were included and uploaded on Rayyan.^12^ Duplicates were removed. All study designs including randomized controlled trials and feasibility studies were included by two independent reviewers (CYKK, AS), provided they were in the English language and abstracts provided sufficient information. AI models were classified into the following: instrument detection, force-based models, eye and instrument tracking.

### Data extraction and quality assessment

Upon initial screening for articles that align with the inclusion criteria, the full text of the selected articles were assessed by two independent reviewers (CYKK, AS). Articles of potential relevance on the reference lists, that were not found through the search method were subjected to the same screening process. Quality of each study was assessed using the Risk of Bias in Non-randomized Studies of Interventions (ROBINS-I) tool by two independent reviewers (CYKK, AS), evaluating the bias in results due to confounding, selection of participants, classification of intervention, deviations from intended interventions, missing data, measurement of outcomes and selection of reported results. The two independent reviewers (CYKK, AS) discussed and addressed data discrepancies.

### Data analysis

Selected data were extracted from each study by two reviewers (CYKK, AS), including the study characteristics, participant demographics, name of AI model, instruments involved, training applications, performance metrics and key findings. Participant demographics such as plastic surgery trainees, details of the AI intervention such as neural network architecture, machine learning algorithms, and training outcomes were extracted. Outcomes related to motion parameters and skill acquisition such as economy of movement, tremor amplitude and flow were recorded. Key characteristics of AI systems including metrics for eye tracking, instrument tracking and detection were noted.

## Results

**Figure 2:**
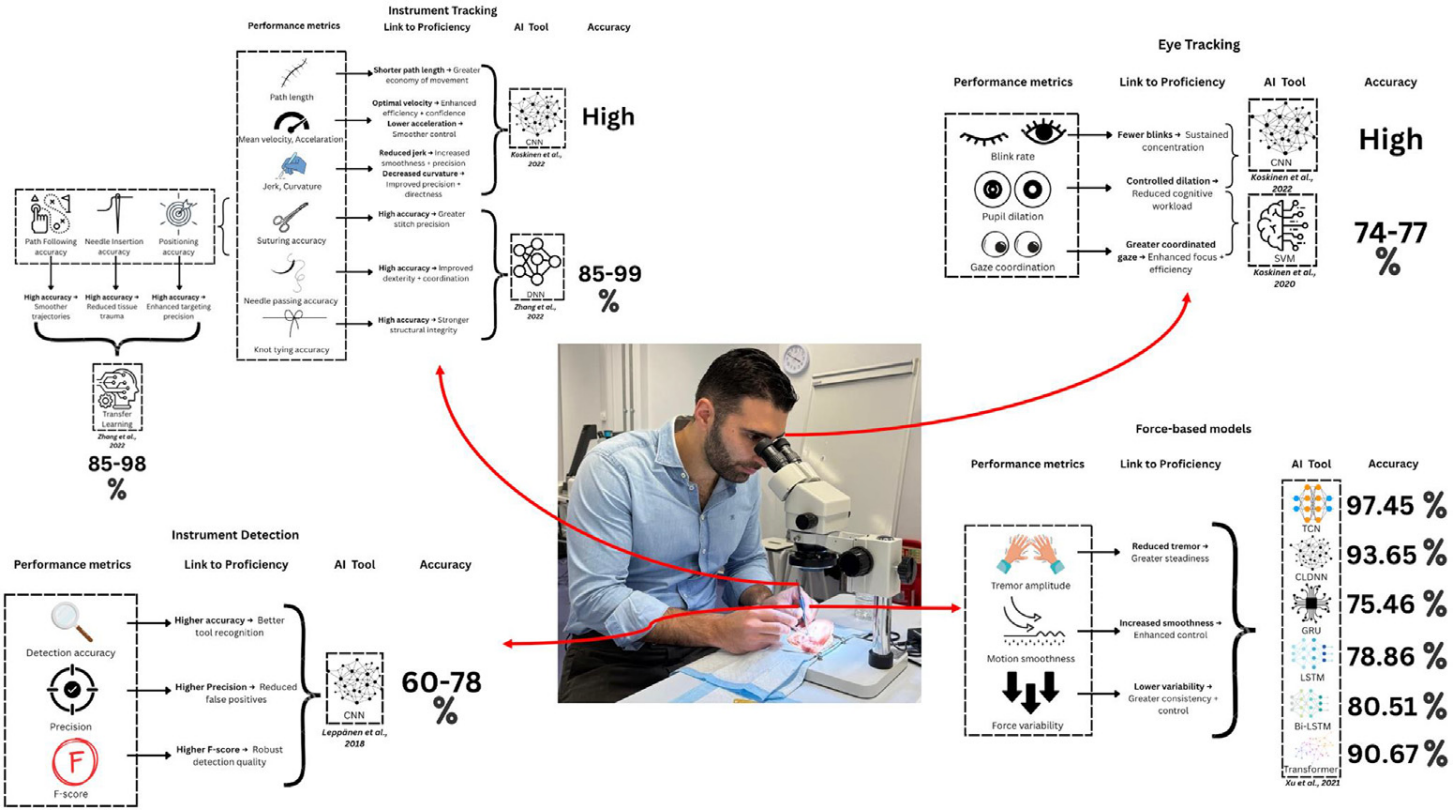
Applications of AI models in the pre-clinical setting.

From the original 499 retrieved articles, 328 abstracts were eligible for screening and upon review of 11 full-text articles, five studies met the inclusion criteria. Table 1 shows an overview of the applications and efficacies of the AI models in microsurgery training. Table 2 shows an overview of the characteristics, mechanisms and applications of the machine learning methods mentioned in this systematic review. Figure 2 is a summary of performance metrics, illustrating of the relationship between human behavior such as eye, hand and instrument movements and proficiency in microsurgery.

**Table 1:** Applications of artificial intelligence to microsurgical training.

AI models	Study	Population and tools	Training applications	Performance metrics	Key findings and contributions
Deep Learning, Eye and Instrument Tracking (CNN)	*Automated Tool Detection with Deep Learning for Monitoring Kinematics and Eye-Hand Coordination in Microsurgery* (Koskinen et al., 2022), Feasibility Study	Dataset: 7500 frames from 20 videos Tools: Microforceps, needle, needleholder, microscissors, suction, drill, hook, Kerrison, knot/loop, scalpel, water irrigation, clamp applier, clamp, dissector, bipolar, aspirator, retractor	Automated tool detection Dual tracking of eye movements and instrument kinematics Monitoring kinematics and eye-hand coordination with real-time analysis Eliminate manual annotations	Normal path length: 0.073–0.257 Normal mean velocity: 0.254–0.864 Normal mean acceleration: 0.238–0.706 Normal mean jerk: 0.258–0.819 Normal mean curvature: 0.594–0.727 Statistical differences between actions (*p* < 0.001) Eye tracking metrics: Pupil dilation and gaze coordination Tool detection accuracy: High (specific metrics not provided)	Developed a CNN-based tool detection model Eliminated manual annotations and external sensors Demonstrated feasibility of real-time tool tracking and kinematics analysis Highlighted transfer learning for generalization across diverse surgical settings
Instrument Tracking, Transfer Learning	*Automatic Microsurgical Skill Assessment Based on Cross-Domain Transfer Learning* (Zhang et al., 2020), Experimental Study	Dataset: Kinematic data from robotic microsurgical tools Tools: Sutures, needle holder, forceps, threads, nanomanipulator, microscopes, master control console	Adapting pre-trained models to classify skill levels Addressing data scarcity issues with transfer learning Automating skill assessment	Suturing accuracy for Deep Neural Network Model: 99.17 % Suturing (Path Following) accuracy for Transfer Learning: 84.72 % Suturing (Positioning) accuracy for Transfer Learning: 97.92 % Suturing (Needle Insertion) accuracy for Transfer Learning: 88.89 % Needle passing accuracy for Deep Neural Network Model: 90.97 % Knot tying accuracy for Deep Neural Network Model: 84.72 %	Demonstrated high accuracy in skill classification using transfer learning Highlighted the effectiveness of adapting pre-trained models to microsurgical tasks Addressed challenges associated with data scarcity in training AI models
Force-Based Models (TCN, CLDNN)	*A Deep Learning Approach to Classify Surgical Skill in Microsurgery Using Force Data from a Novel Sensorised Surgical Glove* (Xu et al., 2023), Feasibility Study	Dataset: 236 trials from 13 surgeons Tools: Sensorized surgical glove with piezoresistive foam sensor mounted on thumb, microscissors, forceps	Force variability analysis Skill classification based on force data Real-time feedback on motion smoothness	Accuracy (random split cross-validation): TCN: 97.45 ± 3.63 %, CLDNN: 93.65 ± 5.39 %, GRU: 75.46 ± 5.05 %, LSTM: 78.86 ± 7.40 %, Bi-LSTM: 80.51 ± 4.06 %, Transformer: 90.67 ± 3.20 % Precision (random split cross-validation): TCN: 97.54 ± 3.70 %, CLDNN: 92.63 ± 6.84 %, GRU: 73.96 ± 9.74 %, LSTM: 88.58 ± 16.58 %, Bi-LSTM: 87.45 ± 7.49 %, Transformer: 94.71 ± 6.21 % Accuracy (LOUO cross-validation): TCN: 88.95 ± 14.80 %, CLDNN: 96.19 ± 3.22 %, GRU: 77.57 ± 10.88 %, LSTM: 76.67 ± 14.33 %, Bi-LSTM: 84.92 ± 9.94 %, Transformer: 86.68 ± 13.37 % Precision (LOUO cross-validation): TCN: 90.00 ± 18.26 %, CLDNN: 98.33 ± 3.73 %, GRU: 78.62 ± 13.30 %, LSTM: 78.50 ± 14.50 %, Bi-LSTM: 90.39 ± 11.34 %, Transformer: 88.20 ± 16.11 % Precision and recall metrics: Not specified	Showed force variability as a key indicator of skill level Demonstrated the superiority of TCN and CLDNN architectures in skill classification compared to GRU and LSTM models Bi-LSTM architecture significantly enhances performance in the LOUO scheme, highlighting the importance of bidirectional data flow in capturing long-range dependencies within force data Transformer network outperforms the GRU, LSTM, and Bi-LSTM models, but is worse than the TCN and the CLDNN in all but one metric (precision). Trans-former’s performance appears to be limited by data scarcity, aligning with the established understanding that Transformers benefit from larger datasets Enabled real-time feedback for tremor amplitude and motion smoothness Highlighted force-based metrics as essential for reducing surgical errors
Eye Tracking (SVM)	*Combined Gaze Metrics as Stress-Sensitive Indicators of Microsurgical Proficiency* (Koskinen et al., 2020), Experimental Study	11 Participants: Novices and experts Tools: Eye-tracking sensors, microscissors, forceps, needle holders	Classification of expertise based on percentage changes in pupil size and blink metrics using a support vector machine classifier Classifier performance was evaluated at segment and suture levels. At the segment level, the classifier was given features from individual segments, and at the suture level, the classifier was given features from all the segments that make up that suture. Monitoring task-related stress and cognitive workload	Accuracy (Segment-Based Classification of Expertise): Needle Pick: 55.5 %, Edge Touch: 66.3 %, Pierce: 54.0 %, Needle Push: 55.1 %, Extraction: 55.1 %, Thread Handling: 53.9 %, Knot 1: 61.4 %, Knot 2: 53.9 %, Knot 3: 59.7 %, Cut: 62.4 % Precision (Segment-Based Classification of Expertise): Needle Pick: 55.6 %, Edge Touch: 64.7 %, Pierce: 54.7 %, Needle Push: 55.1 %, Extraction: 55.1 %, Thread Handling: 54.5 %, Knot 1: 60.6 %, Knot 2: 54.6 %, Knot 3: 58.8 %, Cut: 60.9 % Accuracy (Suture-Based Classification of Expertise with PCA Applied): 76.7 %, 74.7 %, 74.4 %, 73.8 % Precision (Suture-Based Classification of Expertise with PCA Applied): 77.1 %, 74.0 %, 74.7 %, 74.5 % Accuracy (Suture-Based Classification – One Participant Left Out): 72.3 %, 72.0 %, 79.2 %, 74.0 %, 76.1 %, 75.9 %, 76.7 %, 77.7 %, 77.3 %, 77.1 % Precision (Suture-Based Classification – One Participant Left Out): 76.9 %, 69.4 %, 79.4 %, 76.9 %, 76.3 %, 77.3 %, 79.0 %, 78.6 %, 76.9 %, 79.4 % Blink rate contributed less significantly to classification	Pupil dilation is a reliable indicator of cognitive workload and surgical proficiency Eye tracking is able to reflect task-related stress Proposed the use of eye tracking for real-time feedback and stress monitoring during training
Instrument detection (CNN)	*Augmenting Microsurgical Training: Microsurgical Instrument Detection Using Convolutional Neural Networks* (Leppänen et al., 2018), Feasibility Study	Dataset: Surgical videos Tools: Zeiss OPMI and ZeissPico, micro forceps, suture needle, microscissors	Detecting and localizing surgical tools Monitoring instrument motion during training sessions	Accuracy (Detection Rates on Testing Sets): E1 CNN1: 67.3 %, E2 CNN1: 60.1 %, E3 CNN2: 78.3 % Precision (Detection Rates on Testing Sets): E1 CNN1: 65.0 %, E2 CNN1: 60.0 %, E3 CNN2: 73.5 % F-Score (Detection Rates on Testing Sets): E1 CNN1: 78.4 %, E2 CNN1: 75.0 %, E3 CNN2: 84.6 % Processing Speed (fps) (Detection Rates on Testing Sets): E1 CNN1: 8.864, E2 CNN1: 0.066, E3 CNN2: 15.186	High Accuracy and Robust Performance: CNN achieved strong detection rates across different testing sets, with accuracy, precision, and F-score metrics highlighting its reliability. The system enabled precise monitoring of tool motion and positioning, aiding in skill assessment and workflow optimization. CNN-based detection system lays the groundwork for enhanced training environments, where microsurgery trainees can learn from real-time instrument augmentation in recorded and live procedures. The study highlights the potential of transfer learning, multi-object deep learning, and end-to-end learning to improve multi-instrument detection and real-time surgical guidance.

**Table 2:** Overview of machine learning methods.

Machine learning method	Definition and explanation	Application in microsurgery and supermicrosurgery training
Long Short-Term Memory (LSTM)	• Type of recurrent neural network (RNN) that analyze sequences (e.g. video or force data over time) remember long-term dependencies in sequential data. • Mimics human memory by “forgetting” irrelevant details (e.g., minor hand tremors) and retaining important patterns (e.g., consistent suture motions). • Uses “gates” to control what information is stored or forgotten over time.^13^	Force-based skill classification (e.g., analyzing force variability in surgical gloves to assess expertise).^12^
Gated Recurrent Unit (GRU)	• Update gate decides what to keep from past data, while the reset gate discards useless information. • Faster to train while still capturing sequential patterns but it’s less precise for very long sequences.^14^, ^15^	Force-based skill classification (e.g., evaluating surgical glove force data to distinguish novice vs. expert surgeons).^12^
Temporal Convolutional Networks (TCN)	• Neural network using dilated convolutions to analyze time-series data, capturing long-range dependencies. • Analyses the entire sequence at once thus, it is faster and more accurate at spotting long-term trends (e.g. repeated errors in instrument handling).^16^	Achieved highest accuracy (97.46 %) in classifying microsurgical skill levels using force data from sensorized gloves.^12^
Convolutional Long Short-Term Deep Neural Network (CLDNN)	• Combines CNNs for spatial features and LSTMs for temporal patterns to process sequential data with spatial hierarchies (e.g., video). • CNNs extract spatial details (e.g., instrument shapes and location), then LSTMs analyze how those changes over time (e.g. instrument motion during suturing).^17^	Skill classification in microsurgery by combining force-data and temporal motion patterns, it outperforms standalone LSTMs/CNNs in force-data analysis.^12^
Transfer Learning	• Leverages knowledge from a large, general dataset to improve performance on a smaller, specific dataset, often using Deep Neural Network (DNNs) as its foundational programme. • Retains early layers for generic features (e.g. motion patterns) and adapting later layers to the specific domain (e.g. microsurgery). • Reduces training time and data needs, enhances accuracy with small datasets.^18^	Applied to classify skill levels using kinematic data from robotic microsurgical toos, achieving high accuracy (e.g. suturing 99.17 %, positioning 97.92 %) by adapting a pre-trained DNN, addressing data scarcity in microsurgery training.^18^
Support Vector Machine (SVM)	• Finds the optimal boundary to separate data into categories (e.g. novices and experts). • Effective for small datasets by focusing on the most critical data points (e.g. pupil dilation during complex tasks) and high-dimensional data.^19^	Eye-tracking to classify expertise (74.3–76.0 % accuracy using pupil dilation/blink metrics).^20^
You Only Look Once (YOLOv2)	• Real-time object detection algorithm that processes images or videos in a single pass to detect objects and their positions, balancing speed and accuracy. • Uses bounding boxes to track movement and calculate performance metrics (e.g. speed, jerkiness).^21^	Tracking surgical instrument tips to measure procedural time, path distance, and motion smoothness (e.g., jerk index).^22^
Principal Component Analysis (PCA)	• Dimensionality reduction technique that simplifies complex data by identifying key trends. • E.g. It reduces 100 eye-tracking metrics to a few main patterns such as gaze shifts and focus stability for ease of analysis.^23^	Used in eye-tracking studies to reduce feature dimensionality, highlight metrics most linked to skill level and improve classification of surgical proficiency.^20^

## AI models

### Force-based skill classification

Xu et al. developed force-based automated skill classification datasets using a novel sensorized surgical glove. A benchmark dataset comprising 236 trials from 13 surgeons and implemented six deep learning architectures, including Long Short-Term Memory (LSTM), Gate Recurrent Unit (GRU), Temporal Convolutional Networks (TCN), and Convolutional Long Short-Term Deep Neural Network (CLDNN) was established. The TCN achieved the highest accuracy of 97.46 % using random split cross-validation, while the CLDNN performed best in the Leave-One-User-Out (LOUO) scheme with an accuracy of 96.48 %.^24^ Force variability has been a distinguishing factor between novice and expert surgeons, supported by a study by Horeman et al.^20^ on the applicability of the surgical glove force data as an indicator of microsurgical skill.

### Eye tracking for proficiency and workload assessment

Two studies investigated the use of eye tracking to assess microsurgical proficiency and cognitive workload, demonstrating its potential as an objective and scalable evaluation tool. Koskinen et al. explored the use of pupil dilation and blink metrics to classify expertise during standardized suturing tasks.^25^ A support vector machine (SVM) classifier achieved accuracies between 74.3 % and 76.0 % in distinguishing expert and novice surgeons based on pupil size metrics, while blink rate contributed less significantly due to its low occurrence during microsurgery. Pupil dilation effectively reflected task-related stress, cognitive workload and surgical expertise, making it a valuable metric for real-time feedback during surgical training.

In addition to classification, Koskinen et al. combined eye tracking with instrument detection to monitor instrument kinematics and gaze coordination.^18^ Using a convolutional neural network (CNN) trained on 7500 frames from 20 videos of simulated and real surgical procedures, 17 microsurgical instruments were tracked whilst gaze-instrument coordination were analyzed. This real-time, dual-tracking approach eliminated the need for manual annotations or additional kinematic sensors, allowing for efficient evaluation of hand-eye coordination and therefore, improvements in skill acquisition and workflow efficiency in microsurgical training.

Standardized suturing tasks employed by Koskinen et al. involved segmenting the suturing process namely, needle pick, edge touch, piercing, and cutting into phases.^18^ While segment-level classification achieved limited accuracy with needle pick at 55.5 % and cutting at 62.4 %, suture-level classification significantly improved performance, achieving an overall accuracy of 74.3 %–76.0 %. Dimensionality reduction techniques such as principal component analysis (PCA) were applied to enhance model performance and therefore, training outcomes.

Given the ability to monitor task-related stress and performance in real time, eye tracking parameters can act as an objective measure for microsurgical proficiency and cognitive workload, highlighting its potential to enhance the provision of constructive, personalized feedback during training. As such, making the steep learning curve more manageable to the beginner microsurgeon.

### Instrument tracking

Applications of AI to instrument tracking in microsurgical training were demonstrated in four studies. Zhang et al. used transfer learning for skill level classification during robot-assisted microsurgery, achieving high accuracy across tasks such as suturing with an accuracy of 99.17 % and needle passing with an accuracy of 90.97 % using kinematic data from robotic manipulators.^26^ The study highlighted the effectiveness of adapting pre-trained models to microsurgical tasks therefore, addressing data scarcity challenges. In addition, Leppanen et al. implemented a convolutional neural network (CNN) for detecting instruments in surgical videos with successes in localizing and classifying microsurgical instruments, enabling precise monitoring and guidance on instrument movements during practice.^27^ Similarly, Koskinen et al.^18^ combined instrument tracking with eye tracking to analyze instrument kinematics and gaze coordination, demonstrating the feasibility of automated content extraction and analysis for a global assessment of bimanual instrument handling.

### Instrument detection

Leppänen et al.^27^ implemented a Convolutional Neural Network (CNN) to detect and localize surgical instruments in microsurgical training videos, using tools such as Zeiss OPMI and ZeissPico microscopes, micro forceps, suture needles, and microscissors. The system achieved robust detection rates, with the E3 CNN2 configuration scoring an accuracy of 78.3 %, precision of 73.5 %, and F-score of 84.6 %, alongside a processing speed of 15.186 frames per second, demonstrating its capability for real-time monitoring. This focus on static identification and localization contrasts slightly with Xu et al.’s force-based approach, which tracked kinematic variability (e.g., motion smoothness) using a sensorized glove, achieving higher accuracies (TCN: 97.45 %) by analyzing dynamic force data rather than visual detection alone.^24^ Leppänen et al.^27^ CNN system lays the groundwork for augmenting training environments with precise tool recognition, complementing kinematic tracking by providing a foundational step for motion analysis.

### Summary of performance metrics

Across the studies, AI models demonstrated robust performance in classifying surgical skill levels. Force-based models achieved accuracies exceeding 95 %, with TCN and CLDNN emerging as the top-performing architectures. Eye tracking metrics, particularly pupil dilation, showed moderate accuracy in distinguishing experts from novices, while instrument tracking and detection using CNNs and transfer learning achieved high precision in localizing tools and assessing kinematics. Hand motion tracking systems provided valuable feedback on dexterity and movement quality, highlighting their utility in skill refinement.

Overall, the included studies underscore the potential of AI-driven approaches in providing objective, scalable, and real-time feedback for microsurgical training. These technologies offer promising avenues for improving educational outcomes and advancing skill acquisition in microsurgery. Notably, none of the included studies specifically addressed AI applications in supermicrosurgery training, highlighting a gap in the current literature. See Figure 1. All studies demonstrated a moderate risk of bias upon risk of bias assessment using the ROBINS-I tool.

## Discussion

The results of this systematic review demonstrate the significant potential for AI to enhance microsurgery and supermicrosurgery training. AI-driven systems such as motion tracking, deep learning models and computer vision algorithms objectively assess microsurgical skills, provide real-time feedback, and identify areas for improvement.^18^, ^24^, ^25^, ^26^, ^27^, ^28^ Machine, transfer and deep learning have been well-established tool for wider training in robotic, laparoscopic and open surgery with accurate kinematic evaluations improving performance outcomes, with the adaptability of AI models, they are largely applicable to microsurgery training within plastic surgery.^7^^,^^22^, ^26^, ^29^, ^30^, ^31^, ^32^ The quantitative performance metrics formulated by these AI models could complement existing simulation-based training models by offering actionable feedback on the kinematics of microsurgery namely, economy of motion, tremor amplitude and instrument path accuracy, of which are critical for evaluating technical competence in microsurgery. These advancements represent a significant shift from subjective, manual assessments to data-driven, standardized and reproducible evaluation models.

### The impact of AI

The challenges of mastering microsurgical skills using simulation-based training models and manual assessments include subjectivity in assessment results and feedback mechanisms limited to trainer availability and time constraints. Manual grading scales such as the Stanford Microsurgery and Resident Training (SMaRT) scale are prone to inter-rater variability and significant time and expertise are required for implementation.^33^, ^34^, ^35^ Earlier technological attempts to address these issues, such as Rappel et al.’s digital stereo microscope platform, McGoldrick et al.’s video motion analysis system and Franco-Gonzalez et al.’s mitracks 3D stereoscopic tracking system, introduced objective motion tracking to assess dexterity, economy of movement, and bimanual coordination in microsurgery training.^28^^,^^36^^,^^37^ These systems, while innovative for their time, relied on predefined algorithmic measures (e.g., tremor amplitude, path length) and statistical analysis rather than adaptive AI model. AI-driven systems offer standardized evaluations that bridge the gap between subjective assessments and measurable outcomes across training in various surgical specialties and associated skills. For example, Sugiyama et al. developed a deep learning model using You Only Look Once version 2 (YOLOv2) tracked instrumental tips with high precision as evidenced by quantifiable measurements of procedural time, path distance, and normalized jerk index, correlating strongly with traditional skill scales.^32^ Similarly, Xu et al.^24^ demonstrated that force-based models using Temporal Convolutional Networks (TCN) and Convolutional Long Short-Term Deep Neural Networks (CLDNN) could achieve over 95 % accuracy in classifying microsurgical skill levels, outperforming traditional methods reliant on manual, subjective evaluations of proficiency. These AI advancements build on the foundational objectivity of non-AI systems like those of Rappel et al., McGoldrick et al., and Franco-González et al., enhancing automation and adaptability to revolutionize training efficiency in plastic surgery.^28^^,^^36^^,^^37^

### Challenges to using AI in microsurgery training

AI reduces the learning curve of microsurgical skill acquisition, receiving huge interest from the trainee population. However, given the financial, ethical, legal and bureaucratic hurdles associated with the implementation of AI as part of the formal curriculum, delays are likely.^5^ Although the cost-efficiency of using AI remains largely unexplored, these models are likely costly adjuncts to the pre-existing educational resources namely, simulation models and real-time human feedback, considering the immense amount of funding required for research, development and implementation of a user-friendly, intuitive and efficient software.^38^

Despite the demonstrated potential for AI to enhance the microsurgery learning experience, the positive effects of the encouragement, emotional support, motivation and inspiration from a trainer alongside the development of a strong rapport are human factors that are difficult to replicate with AI. Given the data-driven focus of AI models, trainees may experience mental hardship when improvements in the measured outcomes are slow and misaligned with their personal expectations of their ideal progress, highlighting the importance of a holistic curricula in ensuring that a trainee’s emotional well-being, technical skills and personal and career development progress equally well.

### AI application in supermicrosurgery

Supermicrosurgery requires a higher level of eye-microscope-hand coordination to handle vessels with a diameter of 0.3–0.8 mm, which makes extensive training, currently in the form of simulation-based training, essential to the development of technical and psychomotor skill acquisition.^39^ Robotic-assisted surgery (RAS) has demonstrated potential in successful navigation of delicate structures less than 1 mm by completely eliminating ordinary hand tremors for improved precision of movements and widening access to various operating angles given the flexibility of RAS in accessing all anatomical areas.^13^ Although supermicrosurgery represents a technical extension of microsurgery, none of the included studies specifically evaluated AI models in the context of supermicrosurgery. The unique challenges of managing vessels smaller than 0.8 mm and using finer instruments underscore the need for further research directly addressing AI applications in this field.

### Limitations

The papers included in this systematic review have several limitations.^18^, ^24^, ^25^, ^26^, ^27^ Most studies were proof-of-concept or feasibility studies with small sample sizes, limiting the generalizability of findings. In addition, the heterogeneity of methodologies, experimental design, AI architectures and evaluation metrics across studies complicates data synthesis and underscores the need for standardized AI protocols in microsurgical training. For instance, while AI-driven studies like Koskinen et al. employed convolutional neural networks for real-time tool detection, earlier non-AI approaches such as Rappel et al.’s stereo microscope system, McGoldrick et al.’s motion analysis tool, and Franco-González et al.’s mitracks3D used algorithmic motion tracking without machine learning, reflecting a methodological evolution toward AI but also highlighting variability in analytical approaches.^18^^,^^28^^,^^36^^,^^37^ To achieve fair data comparison, the different AI architectures require standardized evaluation of AI performance in microsurgical training to ensure robust validation and reproducibility across studies, of which is yet to be created.

The risk of biases in participant selection were high due to participants being recruited from a small pool of trainees, reducing diversity in participant demographics, skill levels, and geographic settings thus, limiting the applicability of findings to the wider population. Reliance on single-modality data (e.g., video or force sensors) may fail to capture the full complexity of microsurgical tasks. Multimodal data integration, combining kinematic, visual, and physiological metrics, could provide a more comprehensive assessment, however, few studies have explored this approach. The kinematic datasets from non-AI systems such as Rappel et al., McGoldrick et al., and Franco-González et al. could serve as valuable inputs for future multimodal AI models, enhancing their capability to reflect real-world surgical variability.^28^^,^^36^^,^^37^ Although simulation training models are valuable for training and trialing AI models, factors such as the surrounding environment and unpredictable tissue behavior may not fully represent live surgery. Lastly, all studies did not explicitly address the ethical and practical implications of deploying AI in surgical training, including data privacy, algorithm transparency, and trainee reliance on AI-assisted feedback.

## Conclusion

This is the first systematic review to provide a comprehensive evaluation of AI applications in microsurgery and supermicrosurgery training, highlighting their potential to transform plastic surgery education. AI-driven models, such as motion tracking systems, cross-domain transfer learning, deep learning algorithms, and computer vision tools, offer objective and scalable solutions for skill assessment and training optimization. These technologies address critical limitations of traditional methods by providing real-time feedback, reducing reliance on expert evaluators, and enabling personalized training pathways. AI applications beyond motion analysis including vessel handling, vessel flow assessment, integration of virtual reality into training curricula and validation of AI tools in supermicrosurgery-specific tasks could be further explored. Future research should prioritize larger, multicenter studies with standardized protocols for AI model validation, conduct longitudinal studies to assess the long-term impact of AI training, and explore real-world validation to enhance the reliability and impact of AI applications in microsurgery and supermicrosurgery training
